# Associations between physical activity, sedentary time and cardiovascular risk factors among Dutch children

**DOI:** 10.1371/journal.pone.0256448

**Published:** 2021-08-27

**Authors:** Gabrielle ten Velde, Guy Plasqui, Maartje Willeboordse, Bjorn Winkens, Anita Vreugdenhil

**Affiliations:** 1 Department of Pediatrics, Maastricht University Medical Centre, School of Nutrition and Translational Research in Metabolism (NUTRIM), Maastricht, The Netherlands; 2 Department of Nutrition and Movement Sciences, School of Nutrition and Translational Research in Metabolism (NUTRIM), Maastricht University, Maastricht, The Netherlands; 3 Department of Family Medicine, Care and Public Health Research Institute (CAPHRI), Maastricht University, Maastricht, The Netherlands; 4 Department of Methodology and Statistics, Care and Public Health Research Institute (CAPHRI), Maastricht University, Maastricht, The Netherlands; Universitat de les Illes Balears, SPAIN

## Abstract

**Introduction:**

Physical activity (PA) plays an important role in the prevention of cardiovascular diseases, especially in children. Previous studies which investigated the role of PA and sedentary time (ST) in cardiovascular disease used different measurements and found inconsistent results. The current study used recommended standardized measures and provides an overview of PA and ST among Dutch primary school children and their associations with cardiovascular risk factors.

**Methods:**

503 children (55% girls, mean age (± SD) 10 ± 1y) were included. PA (total PA, lightPA and moderate to vigorous PA (MVPA)) and ST were measured with the Actigraph GT3X accelerometer. PA in different domains was measured with the BAECKE questionnaire. Cardiovascular risk factors included BMI z-score, waist circumference, blood pressure (z-score) and estimated cardiorespiratory fitness (CRF) as measured with the 20 meter shuttle run test.

**Results:**

Children spent 57 ± 20 min/day (8%) on MVPA and 42% of the children reached the MVPA guideline of 60 min/day. Total PA and MVPA (h/day) were negatively associated with BMI z-score (B = -0.452, p = 0.011) and waist circumference (B = -3.553, p = 0.011) and positively associated with CRF (B = 2.527, p = <0.001). ST was positively associated with BMI z-score (B = 0.108, p = 0.048) and waist circumference (B = 0.920, p = 0.033). No significant associations were found between total PA or PA intensities and blood pressure.

**Conclusion:**

This study used standardized measures of PA and therefore created an accurate overview of PA, ST and their associations with cardiovascular risk factors. PA and ST were associated with BMI z-score, waist circumference and CRF. The findings emphasize the importance of promoting MVPA in children, but also highlight the potential benefits of reducing ST to improve cardiovascular risk factors.

**Trial registration:**

ClinicalTrials.gov NCT03440580.

## Introduction

Cardiovascular diseases (CVD) are currently the leading cause of death worldwide and it is expected that in 2030, CVD will be the major underlying factor in 22.2 million deaths per year [1]. CVDs are mostly diagnosed during adulthood, however, risk factors for CVDs are already identifiable in children [2]. For instance, the cardiovascular risk factor hypertension is prevalent in primary school children with obesity [3]. Therefore the prevention of cardiovascular diseases should begin in early childhood.

Both association and intervention studies have shown that promoting physical activity (PA) plays an important role in the prevention of CVD [2, 4]. In addition, more time spent sedentary, especially recreational screen time, is related to poorer health outcomes [5]. For example, higher duration of screen time (including television viewing) is associated with poorer fitness and cardiometabolic health [5]. Guidelines have been developed to promote PA and to foster health benefits. The World Health Organization recommends children (4–18 years) to accumulate at least 60 min of moderate to vigorous PA (MVPA) daily, preferably including more vigorous intensity activities, and to minimalize sedentary time (ST), especially recreational screen time [6]. These guidelines also recommend bone-strengthening, and muscle-strengthening physical activities at least 3 times a week [6].

Several methods can be used to measure PA and ST. Objective measurements, such as accelerometry, are more accurate to determine the amount of PA and ST compared to subjective methods (i.e. questionnaires) [7, 8]. According to the review of Steene-Johannessen et al. (2020) who only included studies on objective measurements, more than two thirds of European children can be categorized as insufficiently active [9]. The findings also suggest substantial country specific differences in PA and ST [9]. Studies on PA and ST in Dutch children did not met inclusion criteria and were not taken into account in this review [9]. Nevertheless, more recently, several studies evaluated PA and ST among Dutch children [10–13]. For instance, a study of Remmers et al. (2020) showed that Dutch children performed 55 min/day on MVPA during weekend days compared to 44 min/day on weekdays. Furthermore, they showed that PA significantly declined from primary to secondary school [13]. Regarding ST, the amount tracks from childhood into adulthood which could have an impact on adult health [14]. This highlights the importance of promoting PA and reducing ST in early childhood.

Associations between cardiovascular risk factors and objectively measured PA in children aged 5–19 years are described in several reviews [15–21]. For example, 18 of the 22 studies included in the review of Poitras et al. (2016), found a negative association between total PA and adiposity [17]. In general, MVPA was most frequently examined and provided more consistent associations and larger effect sizes in association with cardiovascular risk factors compared to lower intensity PA [16, 17, 20, 21]. In addition, ST is not taken into account in the majority of the reviews. The sole review which investigated the association between ST and cardiovascular risk factors concluded that based on very low to moderate quality evidence, there were no consistent associations between ST and cardiovascular risk factors [5].

Although several reviews investigated the associations between PA and cardiovascular risk factors, it remains difficult to compare outcome variables across studies due to methodological differences [11, 15]. It has been suggested multiple times that research with standardized measures is needed [8, 17, 22, 23]. For instance, the variation in choice of epochs and cut off points among studies resulted in substantial differences in the amount of PA reported [22, 23].

Additional research is needed on PA and ST and their associations with cardiovascular risk factors. In addition, when studying PA and ST, it is not only essential to investigate total PA and PA intensities, it is also important to study specific domains (i.e. school, sport and leisure time) in which PA takes place and to distinguish between weekday and weekend day [24]. This information can provide meaningful insights in the potential for PA improvement and ST reduction as a target for intervention.

The current study aims to assess PA levels and ST in Dutch primary school-aged children using standardized objective measurements, and study the associations between PA and ST with cardiovascular risk factors (i.e. BMI z-score, waist circumference, blood pressure Z-score and cardiorespiratory fitness (CRF)).

## Materials and methods

### Setting and participants

This study presents the baseline data from the BOOSTH study. BOOSTH is an exergame implemented at the school-level and in order to unlock new levels in the online game, a child needs to be physically active (i.e. walk or run) [25]. Children were recruited via sixteen primary schools, located in the province of Limburg (the Netherlands). A trained research team performed the measurements during school hours. The protocol and methods for this study were described elsewhere [25]. Dutch primary school-aged children (age range 7–12 years) were screened for weight, height and blood pressure, and a shuttle run was performed to assess CRF. PA was measured with the ActiGraph GT3X (Actigraph, Corp, USA) accelerometer and the BAECKE questionnaire. Written informed consent was provided from both parents and children older than 12 years. This study received ethical approval from the Maastricht University Medical Centre, Medical Ethics Committee (METC172043/NL64324.068.17).

### Measurements

#### Physical activity

PA was measured with the Actigraph GT3X (Actigraph, Pensacola, FL, USA). This is a triaxial accelerometer, and represents an accurate and reliable method for objectively measuring both the amount and intensity of PA in children and adolescents [8, 26]. The children were asked to wear the accelerometer attached via a waistband on the right hip for 7 consecutive days during waking hours, except during water activities (eg, showering and swimming) and contact sports (eg, judo). Accelerometry data were downloaded with 10-s epochs using the Actilife software (Actigraph). Valid wear time was defined as a minimum of 4 days, with at least 480 minutes per day of recording, including 1 weekend day. Derived data was expressed as mean counts per minute (cpm). To establish time spent in different intensity categories (ST, light PA (LPA) and MVPA) the Internationally recognized cutoff points developed by Everson et al were used [27].

In addition, the self-administered validated BAECKE questionnaire (for children) has been used to assess the amount of habitual PA ranging from 1 (lowest activity) to 5 (highest activity) [28] (S1 File). PA at school, during leisure time and during organized sports were investigated and analyzed according to the BAECKE formula [28]. The work index was replaced by a school index with similar questions (e.g. “When I am in school, I walk. “never/seldom/sometimes/often/all the time?”).

#### Anthropometrics

Height, weight, and waist circumference were measured in duplicate, and the average of both measurements was reported. Subsequently, BMI (weight [kg]/height [m]^2^) was calculated and age- and gender-specific BMI z-scores were calculated based on reference values from a Dutch population (TNO Growth Calculator, TNO) [29]. In addition, weight classification was based on the International Obesity Task Force (IOTF) classification system [30].

#### Cardiovascular risk factors

Blood pressure measurements were performed thrice using an automated sphygmomanometer (Mobil-O-Graph^®^ Revision 4.6 08/2011 I.E.M. GmbH), with 1 minute of rest in between measurements. A mean value was calculated out of these three readings. The mean systolic and diastolic blood pressure values were computed into z-scores using the LMS method [30] and reference values as described by Wühl et al. [31].

CRF of the children was estimated with a maximum multistage 20-meter shuttle run test. The test was performed using a standardized protocol [25]. The maximum oxygen uptake (VO_2max_) (Y, ml/kg/min) was calculated from the speed (X, km/h) corresponding to the last completed stage following the formula of Leger et al. (1988) (speed = 8 + 0.5 stage number) and age (A, years) as follows: Y = 31.025 + 3.238X − 3.248A + 0.1536AX [32].

#### Screen time

Self-reported screen time was reported separately for weekdays and weekend days in hours per day.

### Statistical analysis plan

Numerical variables were expressed in mean ± standard deviation (SD) and categorical ones as percentage, whereby differences between gender (girls versus boys) were assessed using independent-samples t-tests for numerical variables and chi-square tests for categorical variables. Differences between weekday versus weekend day PA and ST were assessed using paired-samples t-test. Multiple linear regression analyses were used with a cardiovascular risk factor (systolic and diastolic blood pressure z-score, BMI z-score, waist circumference and CRF based on VO_2_max value) as the dependent variable and PA (total PA or PA by intensities (LPA, MVPA and ST) or PA domains (school, sport and leisure time)), age and gender as independent variables. In addition, for systolic and diastolic blood pressure z-score and CRF, BMI z-score was also added as an independent covariate in the model. Assumptions were checked using plots (scatterplots for linearity, P-P-plots and histograms for normality, residual plots for homoscedasticity), where Cook’s distance > 1 was used to define influential outliers. Multicollinearity was checked using the variance inflation factor (VIF), where VIF > 10 indicates a collinearity problem. A p-value ≤0.05 was considered statistically significant. All analyses were performed using IBM SPSS Statistics for Windows version 25.0 (IBM Corp., Armonk, NY, USA).

## Results

Sixteen primary schools (classes 5 until 7; aged 7–12 years) were involved in this study. 1159 children were asked to participate in the study of which in total 710 (62%) children participated. On average 45 (range 17–89) children participated per school. Among the 710 recruited children, 503 children had valid wear time data and could be included in the analyses. There was missing data for BMI (z-score) (n = 5), waist circumference (n = 7), systolic blood pressure (n = 7), diastolic blood pressure (n = 12), cardiorespiratory fitness (n = 50) and incomplete information on BAECKE school index (n = 13), BAECKE sport index (n = 22), BAECKE leisure time index (n = 51) and BAECKE total score (n = 59).

### Children characteristics

There were no significant differences in participant characteristics (i.e. age, gender, BMI-z score) between the whole population and children who reached valid wear time.

Description of children’s characteristics are shown in Table 1. The mean (± SD) age was 10 ± 1 years (age range 7–12 years), 45% were boys and 22% of the children were classified as overweight/obese.

**Table 1:** Participant characteristics.

	N	Total Mean ± SD	N	Girls Mean ± SD	N	Boys Mean ± SD	P value
**Demographics**							
Age, years	503	10 ± 1	277	10 ± 1	226	10 ± 1	0.381
Gender, F/M %	503	55/ 45					
**Anthropometry**							
BMI	498	17.9 ± 3.1	274	18.1 ± 3.3	224	17.7 ± 2.8	0.198
BMI z-score	498	0.16 ± 1.06	274	0.17 ± 1.06	224	0.16 ± 1.05	0.879
Waist circumference, cm	496	62.7 ± 8.8	273	62.5 ± 9.1	223	63.00 ± 8.5	0.525
**IOTF**							
Normal weight, %	388	77	209	76	179	79	
Overweight/obese, %	114	23	67	24	47	21	
**Cardiovascular health**							
SBP, mmHg	496	104.6 ± 10.9	274	104.1 ± 11.7	222	105.1 ± 9.7	0.336
SBP z-score	491	-1.1 ± 1.4	271	-1.1 ± 1.5	220	-1.1 ± 1.4	0.556
DBP, mmHg	496	68.8 ± 8.6	274	69.4 ± 9.0	222	68.1 ± 8.1	0.099
DBP z-score	491	-0.5 ± 1.4	271	-0.3 ± 1.5	220	-0.6 ± 1.3	0.053
20m shuttle run, stage	453	5.9 ± 2.2	250	5.5 ± 2.1	203	6.3 ± 2.4	**<0.001**
Estimated CRF ml/kg/min	453	46.7 ± 5.5	250	46.1 ± 5.3	203	47.6 ± 5.6	**0.004**
**Objectively measured PA per day**							
CPM	503	1151 ± 272	277	1129 ± 259	226	1177 ± 286	**0.049**
ST, min/day	503	429 ± 64	277	425 ± 59	226	434 ± 69	0.111
ST, % / day	503	59 ± 7	277	59 ± 7	226	59 ± 7	0.977
LPA, min/day	503	240 ± 39	277	241 ± 39	226	238 ± 39	0.401
LPA, % / day	503	33 ± 5	277	34 ± 5	226	33 ± 5	**0.028**
MVPA, min/day	503	57 ± 20	277	53 ± 17	226	61 ± 21	**<0.001**
MVPA, % / day	503	8 ± 3	277	7 ± 2	226	8 ± 3	**<0.001**
Wear time min/day	503	726 ± 56	277	719 ± 51	226	734 ± 61	**0.004**
MVPA guideline 60 min/day	503	41.6%	277	35.7%	226	48.7%	**0.004**
**PA as measured with BAECKE questionnaire**							
School related PA	490	2.75 ± 0.34	269	2.72 ± 0.32	221	2.80 ± 0.37	**0.011**
Sport related PA	481	2.97 ± 0.67	264	2.86 ± 0.64	217	3.11 ± 0.67	**<0.001**
Leisure time PA	452	3.04 ± 0.62	252	3.06 ± 0.61	200	3.02 ± 0.62	0.548
Total PA	444	8.73 ± 1.19	248	8.62 ± 1.15	196	8.88 ± 1.21	**0.021**
**Screen time**							
Screen time weekday (h/d)	485	3.05 ± 1.98	266	2.67 ± 1.85	219	3.53 ± 2.03	**<0.001**
Screen time weekend day (h/d)	485	3.32 ± 2.09	266	2.97 ± 2.06	219	3.74 ± 2.05	**<0.001**

Abbreviations: BMI, body mass index; CPM, counts per minute; CRF, cardiorespiratory fitness; DBP, diastolic blood pressure; MVPA, moderate to vigorous PA; PA, physical activity; ST, sedentary time; SBP, systolic blood pressure. Notes: Physical activity intensities are presented as mean minutes per day ± standard deviation and as percentage of valid wear time. A bold p-value refers to a statistically significant number considered ≤0.05.

### Description of physical activity levels and sedentary time

PA levels and ST for the whole population as well by gender are shown in Table 1. Children spent on average 240 ± 39 min/day (33%, based on wear-time) on LPA and 57 ± 20 min/day (8%) on MVPA. The percentage of children who reached the PA guideline of a minimum of 60 minutes of MVPA per day was 42% (N = 209). In addition, children spent on average 429 ± 64 min/day in ST which correspondents to 59% of the day. Compared to girls, boys spent on average significantly more time in total PA (difference 48 cpm, p = 0.049), and MVPA (8 min/day, p = <0.001). Furthermore, boys spent more time of the day in LPA (1%/day, p = 0.028) while this was not significant when LPA was expressed in minutes per day. When PA was measured with the BAECKE questionnaire, boys spent on average more time in school related PA, organized sports and total PA. Table 2 shows the differences in PA levels and ST between weekdays and weekend days. Children had significantly more PA (cpm) on a weekend day compared to a week day (79 ± 368 cpm). During a weekend day, children spent significant less time in ST (-38 ± 88 min/day) and LPA (-9 ± 52 min/day), whilst they spend significantly more time in MVPA (3 ± 28 min/day). The average wear time per day was higher on a weekday (45 ± 105 min/day).

**Table 2:** PA patterns during week and weekend day separately (N = 503).

	Weekday Mean ± SD	Weekend day Mean ± SD	P value
**PA**			
CPM	1133 ± 264	1212 ± 436	**<0.001**
ST min/day (%)	440 ± 63 (59)	401 ± 100 (58)	**<0.001**
LPA min/day (%)	243 ± 40 (33)	233 ± 58 (34)	**<0.001**
MVPA min/day (%)	56 ± 19 (8)	59 ± 32 (9)	**0.029**
Wear time min/day	739 ± 57	693 ± 107	**<0.001**

Abbreviations: CPM, counts per minute; MVPA, moderate to vigorous PA; PA, physical activity; ST, sedentary time; VPA, vigorous PA. Notes: Physical activity intensities are presented as mean minutes per day ± standard deviation and as percentage of valid wear time. A bold p-value refers to a statistically significant number considered ≤0.05.

#### Associations between objectively measured PA and cardiovascular risk factors

Total PA (cpm) was negatively associated with BMI z-score (mean change (B) = -0.079 per 100 cpm, p = < 0.001) and waist circumference (B = -0.681 per 100 cpm, p = <0.001), and positively associated with CRF (B = 0.184 per 100 cpm, p = 0.030) (Table 3). Cardiovascular risk factors and their associations with PA intensities are shown in Table 4. No significant associations with cardiovascular risk factors were found for LPA. An increase in MVPA was negatively associated with BMI z s-score and waist circumference (B = -0.452, p = 0.011 versus B = -3.553, p = 0.011 respectively). CRF was positively associated with MVPA (B = 2.527, p = <0.001). ST was positively associated with BMI z-score and waist circumference (B = 0.108, p = 0.048 and B = 0.920, p = 0.033, respectively).

**Table 3:** Associations between total PA (CPM) and cardiovascular risk factors.

	Systolic BP z–score (N = 491) ^b^	Diastolic BP z–score (N = 491) ^b^	BMI z–score (N = 498) ^a^	Waist circumference (N = 496) ^a^	Estimated CRF (N = 453) ^b^
**PA (CPM)**					
Β (95% CI) P value	0.027 (-0.024, 0.077) 0.297	0.003 (-0.047, 0.052) 0.918	-0.079 (-0.115, -0.043) **<0.001**	-0.681 (-0.966, -0.395) **<0.001**	0.184 (0.018, 0.350) **0.030**

Abbreviations: CPM, counts per minute; CRF, cardiorespiratory fitness; CI, confidence interval.

B = mean change in outcome if PA increases with 100 cpm.

^a^ Corrected for age and gender.

^b^ Corrected for age, gender and BMI z-score.

Note: a bold p-value refers to a statistically significant number considered ≤0.05.

**Table 4:** Associations between PA intensities and cardiovascular risk factors.

	Systolic BP z–score (N = 490)^b^ Β (95% CI) P value	Diastolic BP z–score (N = 490) ^b^ Β (95% CI) P value	BMI z—score (N = 496) ^a^ Β (95% CI) P value	Waist circumference (N = 495) ^a^ Β (95% CI) P value	Estimated CRF (N = 452) ^b^ Β (95% CI) P value
ST hour/day	-0.064 (-0.209, 0.082) 0.389	-0.024 (-0.168, 0.120) 0.740	0.108 (0.001, 0.215) **0.048**	0.920 (0.075, 1.764) **0.033**	-0.342 (-0.127, 0.812) 0.152
LPA hour/day	-0.151 (-0.381, 0.078) 0.196	-0.167 (-0.394, 0.060) 0.148	0.099 (-0.070, 0.267) 0.251	0.703 (-0.625, 2.032) 0.299	0.061 (-0.687, 0.810) 0.872
MVPA hour/day	0.224 (-0.252, 0.700) 0.355	0.231 (-0.240, 0.732) 0.336	-0.452 (-0.799, -0.105) **0.011**	-3.553 (-6.281, -0.826) **0.011**	2.527 (0.996, 4.057) **<0.001**

Abbreviations: BP, blood pressure; CRF, cardiorespiratory fitness; LPA, LPA; MVPA, moderate to vigorous PA; PA, physical activity; ST, sedentary time.

B = corrected mean change in outcome if specific PA intensity (ST, LPA or MVPA) increases with 1 hour/day.

^a^ Corrected for age and gender.

^b^ Corrected for age, gender and BMI z-score.

Note: a bold p-value refers to a statistically significant number considered <0.05.

### Associations between subjectively measured PA domains and cardiovascular risk factors

PA during school was positively associated with BMI z-score (B = 0.330, p = 0.034). Sports related PA and PA during leisure time were positively associated with CRF (B 1.019, p = 0.006 and B = 0.802, p = 0.036 respectively). No significant associations were found between any of the specific PA domains (school related PA, organized sports and leisure time) and waist circumference or blood pressure z-scores (S1 Table).

## Discussion

This study investigated PA as well as ST among Dutch children and the associations with cardiovascular risk factors. The present study shows that the majority (58.4%) of the children do not meet the recommended MVPA of 60 minutes per day. Children spent on average 59% of the day sedentary. The amount of time spent in MVPA was higher among boys than girls. Children performed more total PA (cpm) and less ST on a weekend day compared to a week day. Time spent in total PA and MVPA was negatively associated with BMI z-score and waist circumference and positively associated with CRF. ST was positively associated with BMI z-score and waist circumference. No significant associations were found for LPA and any of the cardiovascular risk factors.

The results of the present study regarding ST are comparable with the results of the study of Bartelink et al. (2019) who showed that Dutch primary school children spent around 60 percent of their time sedentary [11]. Specific recommendations on the amount of ST per day are lacking i.e. it is recommended to avoid long periods of sitting [6]. A longitudinal study across childhood and adolescence showed that the amount of time spent sedentary increases as children grow older, especially between 9 and 12 years old [33]. Therefore, interventions need focus on preventing the decrease in overall ST. Specially, interventions aiming to reduce screen-time might be effective, as screen-time contributes the most to leisure-time sedentary behavior among children [34]. The results of the current study showed that children spent around 2.7 to 3.7 hour/day on screen-time. This result is higher compared to a European study, in which children from a similar age range (10–12 years) spent around 2 to 2.5 hours per day on screen-time. This highlights the urgent need for decreasing specific screen-time related ST.

In the present study, boys spent more time in MVPA compared to girls, which is in line with previous studies [11–13]. The higher level of total PA and MVPA in boys could be explained by a higher intrinsic motivation and experiencing more interest and enjoyment from PA compared to girls [35]. Furthermore, boys and girls often have different preferences in regard to PA types and play behavior [36, 37]. For example, boys participate more in sports and active games, while girls prefer walking and/running or play in a playground [37].

Although there is inconsistency in previous literature with regard to the associations between PA, ST and cardiovascular risk factors, in general, the results support a stronger association of MVPA with cardiovascular risk factors compared to LPA or ST [5, 16, 17, 20, 21]. More specific and in line with the findings in this study, previous studies reported that MVPA was negatively associated with BMI z-score and waist circumference, and positively associated with CRF [16, 17, 19–21]. The present study found a positive association between ST and BMI z-score. The review of Carson et al. (2016) showed no statistically significant associations between objectively measured ST and body composition [5]. However, their results suggest that higher screen time duration is associated with unfavorable body composition [5].

Blood pressure (z-score) was not significantly associated with PA or ST in the present study. The findings from previous studies on the relation between blood pressure and PA are contradictory. The review of Garcia et al. (2020) showed that vigorous PA was not associated with systolic blood pressure [20]. The review of Andersen et al. (2011) agreed that there is no clear association between PA and blood pressure in normotensive children, only a positive effect of PA on reducing blood pressure in children with hypertension [18]. On the other hand, the results of the review of Poitras et al. (2016) showed that in the majority of studies total PA was favorably associated with both diastolic blood pressure and systolic blood pressure [17]. Besides the accuracy and choice of methods for PA measurement, the inconsistency in Results may also be due to the initial health status of children in various studies, which may greatly affect the observed associations [16, 20]. For instance, factors known to affect blood pressure among children are age, gender, BMI, race/ethnicity and socioeconomic status [38–41]. Therefore, in the present study, we corrected for age, gender and BMI z-score in the linear regression model. The data show that when these factors are taken into account, there were no associations between blood pressure and PA levels or ST. A possible explanation is that the study population in the present study consist of children that are relatively young (mean age of 10 year) and the majority is classified with a normal body weight (78.1%).

The present study shows that subjectively measured PA during school was positively associated with BMI z-score (B = 0.330, p = 0.034). This is in contrast with the objectively measured results, which show that total PA and MVPA were negatively associated with BMI z-score. The advantage of the use of the BAECKE questionnaire is that PA regarding different domains (school related PA, organized sports and leisure time) is shown. However, the possibility of over- or underestimation of self-reported data cannot be ignored and it is also possible that children may not fully understand the concept of PA, especially for school related PA [8]. For organized sports, it is possible that children provided a more correct amount of PA, since these questions may be more clear and hence reported more accurately (i.e. the amount and time of organized sports per week). It is recommended for future researchers to objectively measure PA (with accelerometry), in combination with the use of a diary to detect the amount of PA during specific domains.

A methodological strength of this study was the use of recommended standardized methods to measure and analyze PA and ST. Previous studies used different methods which could result in considerable differences in the amount of PA and ST [42]. This makes it difficult to compare outcomes between studies. Therefore, the present study used the cut off points of Evenson, which are based on free-living activities of 5–15 year old children [27, 43]. Furthermore, shorter epochs (i.e. 10 or 15 seconds) should better capture the quick changes in PA patterns compared to longer epochs (i.e. 60 seconds), especially in young children [42]. Therefore, the present study used 10 second epochs for downloading the accelerometer data. By the use of standardized methods, it is expected that the results of the present study provide an accurate overview of PA, ST and their associations with cardiovascular risk factors. Furthermore, to our knowledge, this is the first study among Dutch children, investigating the associations between PA, ST and cardiovascular risk factors. Due to country specific differences in PA and ST [9], this study provides valuable information for this specific population. However, a relative small age range (7–12 years) was included in the present study. Previous studies showed that PA levels increase with age, up to an age of 10–11 years old, and then decrease at >11 years when children head into puberty [13, 44, 45]. Future research could recruit children in other age groups and investigate differences between age groups. A limitation of the present study was the use of the GT3X accelerometer to classify ST. According to Janssen et al. (2015) it seems that the ActivPAL is most accurate to measure ST since the activPAL uses the inclination of the thigh to predict time spent lying, sitting, standing, and stepping. However, the ActiGraph GT3X also provide the possibility of classifying posture through the use of equations that estimate inclination from raw triaxial data [8, 42]. The present study did not differentiate in different kind of sedentary behaviors (i.e. sitting, lying). Furthermore, children were instructed to remove the accelerometer during water activities and some contact sports, which may have had an impact on the accelerometry data. The time spent on these activities is generally very small compared to the entire observation interval. It is recommended to use a waterproof accelerometer for future research to ensure that a higher amount of activities would be included in the PA data. In addition, it should be noted that the wear time is higher on a weekday. Non wear time intervals include periods in which children were asked not to wear the accelerometer, such as sleeping. Total sleep time is longer on weekend days [46] and therefore it is likely that children have a lower wear time on weekend days compared to a week day. This might have an effect on the finding that children performed more total PA (cpm) and less ST on a weekend day compared to a week day.

In the present paper, baseline data are described before the start of the (BOOSTH) intervention. Follow-up data of the BOOSTH intervention will demonstrate whether changes in PA levels and ST in the long term have an influence on cardiovascular risk factors among primary school- aged children. Future research and practical implications are to focus especially on promoting MVPA and reducing ST with extra attention towards weekdays and girls gender.

## Conclusion

This cross-sectional study showed that the majority of Dutch children is insufficiently physically active. Fifty-eight percent of the children do not meet the recommended MVPA of 60 minutes per day and children spent on average 59% of the day sedentary. Higher PA levels were associated with several favorable cardiovascular risk factors including a lower BMI, lower waist circumference, and a higher CRF. On the other hand, high levels of ST were associated with unfavorable cardiovascular risk factors including a higher BMI z-score and waist circumference. Blood pressure was not related to either PA or ST. These findings stress the importance of promoting MVPA in children, but also highlight the potential benefits of reducing ST to prevent CVD.

## Supporting information

S1 File Baecke questionnaire—The questionnaire of Baecke et al for measurement of a person’s habitual physical activity. (PDF)

S1 Table Associations between subjectively measured PA domains and cardiovascular risk factors. Abbreviations: BP, blood pressure; CRF, cardiorespiratory fitness. B = corrected mean change in outcome if specific PA domain (school, sport, leisure time) increases. ^a^ Corrected for age and gender. ^b^ Corrected for age, gender and BMI z-score.
(DOCX)

## Abbreviations

BP: blood pressure
CPM: counts per minute
CRF: cardiorespiratory fitness
LPA: light physical activity
MPA: moderate physical activity
MVPA: moderate-to-vigorous physical activity
PA: physical activity
ST: sedentary time
