# Supplementary material for: Associations between physical activity, sedentary time and cardiovascular risk factors among Dutch children
Source: PLoS One. 2021 Aug 27;16(8):e0256448. doi: 10.1371/journal.pone.0256448 (PMC8396731; doi:10.1371/journal.pone.0256448)
Supplement: S1 File — (PDF) [file pone.0256448.s001.pdf]

The Questionnaire of Baecke et al for Measurement of a Person's Habitual Physical Activity

Overview:

Baecke et al developed a questionnaire for evaluating a person's physical activity and separating it into three distinct dimensions. The authors were from the Netherlands.

Indices for physical activity:

(1) work activity

(2) sports activity

(3) leisure activity

Work Index

Question	Response	Points
What is your main occupation?	low activity	1
	moderate activity	3
	high activity	5
At work I sit	never	1
	seldom	2
	sometimes	3
	often	4
	always	5
At work I stand	never	1
	seldom	2
	sometimes	3
	often	4
	always	5
At work I walk	never	1
	seldom	2
	sometimes	3
	often	4
	always	5

At work I lift heavy loads	never	1
	seldom	2
	sometimes	3
	often	4
	always	5
After working I am tired	very often	5
	often	4
	sometimes	3
	seldom	2
	never	1
At work I sweat	very often	5
	often	4
	sometimes	3
	seldom	2
	never	1
In comparison of others of my own age I think my work is physically	much heavier	5
	heavier	4
	as heavy	3
	lighter	2
	much lighter	1

where: • The work activity is according to the Netherlands Nutrition Council with (1) low activity including clerical work driving shopkeeping teaching studying housework medical practice and occupations requiring a university education; (2) middle activity including factory work plumbing carpentry and farming; (3) high activity includes dock work construction work and professional sport.

work index = ((6 – (points for sitting)) + SUM(points for the other 7 parameters)) / 8

Sport Index

Question	Response	Points
Do you play sports?	yes then calculate sport score	(see below)
	• sport score >= 12	5
	• sport score 8 to < 12	4
	• sport score 4 to < 8	3
	• sport score 0.01 to < 4	2
	• sport score = 0	1
	No	1
In comparison with others of my own age I think my physical activity during leisure time is	much more	5
	More	4
	the same	3
	Less	2
	much less	1
During leisure time I sweat	very often	5
	Often	4
	sometimes	3
	Seldom	2
	Never	1
During leisure time I play sport	Never	1
	Seldom	2
	sometimes	3
	Often	4
	very often	5

Data on Most Frequently Played Sport	Finding	Value
What sport do yo play most frequently	low intensity	0.76
	medium intensity	1.26
	high intensity	1.76
How many hours do you play a week?	< 1 hour	0.5
	1-2 hours	1.5
	2-3 hours	2.5
	3-4 hours	3.5
	> 4 hours	4.5
How many months do you play in a year?	< 1 month	0.04
	1-3 months	0.17
	4-6 months	0.42
	7-9 months	0.67
	> 9 months	0.92

where: • The sport intensity is divided into 3 levels: (1) low level (billiards sailing bowling golf etc) with an average energy expenditure of 0.76 MK/h; (2) middle level (badminton cycling dancing swimming tennis) with an average energy expenditure of 1.26 MJ/h; (3) high level (boxing basketball football rugby rowing) with an average energy expenditure of 1.76 MJ/h

**Data on Second Most Frequently Played Sport**	**Finding**	**Value**
What sport do you play most frequently	low intensity	0.76
	medium intensity	1.26
	high intensity	1.76
How many hours do you play a week?	< 1 hour	0.5
	1-2 hours	1.5
	2-3 hours	2.5
	3-4 hours	3.5
	> 4 hours	4.5
How many months do you play in a year?	< 1 month	0.04
	1-3 months	0.17
	4-6 months	0.42
	7-9 months	0.67
	> 9 months	0.92

simple sports score = ((value for intensity of most frequent sport) * (value for weekly time of most frequent sport) * (value for yearly proportion of most frequent sport)) * ((value for intensity of second sport) * (value for weekly time of second sport) * (value for yearly proportion of second sport))

sport index = (SUM(points for all 4 parameters)) / 4

Leisure Index

**Question**	**Response**	**Points**
During leisure time I watch television	never	1
	seldom	2
	sometimes	3
	often	4
	very often	5
During leisure time I walk	never	1
	seldom	2
	sometimes	3
	often	4
	very often	5
During leisure time I cycle	never	1
	seldom	2
	sometimes	3
	often	4
	very often	5
How many minutes do you walk and/or cycle per day to and from work school and shopping?	< 5 minutes	1
	5-15 minutes	2
	15-30 minutes	3
	30-45 minutes	4
	> 45 minutes	5

leisure index = ((6 – (points for television watching)) + SUM(points for remaining 3 items)) / 4

References:

Baecke JAH Burema J Frijters ER. A short questionnaire for the measurement of habitual physical activity in epidemiological studies. Am J Clin Nutr. 1982; 36: 936-942.
