# Supplementary material for: Associations between physical activity, sedentary time and cardiovascular risk factors among Dutch children
Source: PLoS One. 2021 Aug 27;16(8):e0256448. doi: 10.1371/journal.pone.0256448 (PMC8396731; doi:10.1371/journal.pone.0256448)
Supplement: S1 Table — Abbreviations: BP, blood pressure; CRF, cardiorespiratory fitness. B = corrected mean change in outcome if specific PA domain (school, sport, leisure time) increases. a Corrected for age and gender. b Corrected for age, gender and BMI z-score. (DOCX) [file pone.0256448.s002.docx]

|  | **Systolic BP z – score (N=434)** ^b^  Β (95% CI) P value | **Diastolic BP z – score**  **(N=434)** ^b^  Β (95% CI) P value | **BMI z - score**  **(N=440)** ^a^  Β (95% CI) P value | **Waist circumference (N=439)** ^a^  Β (95% CI) P value | **Estimated CRF**  **(N=396)** ^b^  Β (95% CI) P value |
| --- | --- | --- | --- | --- | --- |
| BAECKE school score | 0.048 (-0.374, 0.470) 0.824 | 0.066 (-0.351 ,0.482) 0.757 | 0.330 (0.024 , 0.636) **0.034** | 2.063 (-0.344 , 4.471) 0.093 | 0.215 (-1.150 , 1.579) 0.757 |
| BAECKE sport score | 0.096 (-0.130, 0.323) 0.403 | 0.020 (-0.203 , 0.244) 0.857 | -0.118 (-0.284, 0.047) 0.161 | -1.126 (-2.426 , 0.174) 0.089 | 1.019 (0.292 , 1.746) **0.006** |
| BAECKE leisure time score | 0.099 (-0.137 , 0.335) 0.411 | 0.231 (-0.240 , 0.732) 0.336 | 0.071 (-0.162 , 0.304) 0.548 | 0.634 (-0.708 , 1.976) 0.354 | 0.802 (0.051 , 1.553) **0.036** |

**S1 Table**

**Table 1: Associations between subjectively measured PA domains and cardiovascular risk factors**

Abbreviations: BP, blood pressure; CRF, cardiorespiratory fitness. B = corrected mean change in outcome if specific PA domain (school, sport, leisure time) increases. ^a^ Corrected for age and gender. ^b^ Corrected for age, gender and BMI z-score.
